# Features of the Formation of Sensitive Films Based on Mycelium of Higher Fungi for Surface and Plate Acoustic Waves Gas Sensors

**DOI:** 10.3390/s23042216

**Published:** 2023-02-16

**Authors:** Andrey Smirnov, Vladimir Anisimkin, Larissa Krasnopolskaya, Olga Guliy, Ilya Sinev, Viacheslav Simakov, Alexander Golyshkin, Nailya Almyasheva, Nikita Ageykin, Iren Kuznetsova

**Affiliations:** 1 Kotelnikov Institute of Radio Engineering and Electronics, Russian Academy of Sciences, 125009 Moscow, Russia; 2 Gauze Institute of New Antibiotics, 119021 Moscow, Russia; 3 Institute of Biochemistry and Physiology of Plants and Microorganisms—Subdivision of the Federal State Budgetary Research Institution Saratov Federal Scientific Centre of the Russian Academy of Sciences (IBPPM RAS), 410049 Saratov, Russia; 4 Department of Material Sciences and Technologies and Quality Management, Institute of Physics, Saratov State University Named after N.G. Chernyshevsky, 410012 Saratov, Russia

**Keywords:** mycelium film, *Ganoderma lucidum*, delay line, SAW, plate acoustic wave, acoustic gas sensor

## Abstract

A comparative analysis of the responses of two types of acoustic waves (surface SAW and plate APW) with close frequencies and the same type of waves (SAW) with different frequencies toward various liquid vapors (water, acetone, ethanol) was carried out in this paper. Two types of films based on mycelium of higher fungus *Ganoderma lucidum* (Curtis) P. Karst (*G. lucidum*) prepared by various methods were used as sensitive coatings. These films were based on *G. lucidum* mycelium ethanolic (48% *v*/*v*) homogenizate (MEGl) and extract (EGl). A film deposition procedure compatible with acoustic devices technology was developed. Various piezoelectric substrates (YX-LiNbO_3_, 128 YX-LiNbO_3_) were used for appropriate acoustic delay lines production. It was found that additional SAW and APW attenuation associated with the appearance of mycelium films on the surface of the acoustic waveguide is two times greater for MEGL than for EGL films in the frequency range of 20–80 MHz The changes in acoustic wave amplitude and phase due to vapor absorption were measured and compared with each other, taking into account the differences in geometry of the samples. It was found that the phase response of the SAW delay lines with EGL films is three times higher than one with the presence of MEGL films for water and ethanol vapors. The films used are demonstrated good reproducibility and long-term stability for at least 2 months. Based on the results obtained, it was concluded that MEGl film is not appropriate for use in high frequency SAW delay lines as a sensitive coating. However, both types of the films (MEGl and EGl) could be used as sensitive coatings for low frequency SAW and APW sensors based on corresponding delay lines. Additionally, it was found that the films used are not sensitive to acetone vapor. As a result of the work carried out, a technique for creating sensitive films based on the mycelium of higher fungi compatible with the planar technology of acoustoelectronic delay lines was developed. The possibility of using such films for the development of gas SAW and APW sensors was shown.

## 1. Introduction

Currently, there are a large number of works devoted to the development of acoustic gas sensors [1]. The principle of their operation is based on the using either the anisotropy of the substrate [2], a sensitive film [3], or special designs of acoustic devices [3,4,5]. The interaction of the sensitive film with the environment leads to a change of its physical characteristics (conductivity, permittivity, viscosity, elastic moduli, or density). A change of the film properties, in turn, leads to a change of the characteristics of acoustic signals (frequency, phase, and amplitude) in the acoustoelectronic devices. This is possible as a result of acoustic contact between a sensitive film and the piezoelectric substrate. One of the main tasks in the development of acoustic chemical sensors is the search for new promising sensor coatings [6,7]. These coatings should be cheap, selective, environmentally friendly, and technologically advanced for the manufacturing of sensors. Such materials should be easily recyclable and leave no carbon footprint [8]. Modern sensitive materials are included various coatings obtained by means of biotechnological approaches [9,10,11]. One of such approach is using mycelium of higher fungi. The kingdom of higher fungi contains a huge amount of mushroom types. It is known that various fungi are capable of absorbing chlorines, heavy metals, and chemical components of car exhaust gases [12,13]. It has recently been shown that films based on mycelium of the *Lentinula Edodes* strain F-249 can be used as selective sensor coatings for a bulk acoustic wave (BAW) resonator [14]. These films reacted selectively to ammonia, formaldehyde, and ethyl acetate vapors, depending on the method of their cultivation and preparation. It also was shown that the using a film based on the extract from *Pleurotis ostreatis* makes it possible to implement a bulk acoustic wave (BAW) resonator sensor with high sensitivity to phenol vapor [15]. Further studies have shown that mycelium films can be used to create reusable acoustic humidity sensors [16]. It also was shown that mycelium films of higher fungi with thicknesses up to 1 µm can be used as sensor coatings for BAW resonators with an operating frequency up to 3.5 GHz [17]. However, the studies carried out are insufficient for an unambiguous conclusion about the possibility of the development of gas sensors based on acoustic devices with mycelium films as sensitive coatings. It is necessary to carry out further studies on the temporal stability of the devices being created, the durability, the time of response/recovery, the repeatability, the quality of the film obtained, etc.

It should be noted that not only BAW resonators, but also delay lines based on surface (SAW) and plate (APW) acoustic waves can serve as the basis for gas sensors [18,19]. In the case of a BAW resonator sensor, only the changes of the mechanical properties (density, elastic constants, and viscosity) of the sensitive film due to gas-film interaction can be detect. In the case of SAW or APW delay line based on piezoelectric substrate, the changes are not only mechanical, but also the electric properties (conductivity, permittivity) of the sensitive coating due to the gas–film interaction can be fixed.

A sensitive film must be placed between the interdigital transducers (IDTs) in the case of using SAW and APW delay lines. Moreover, the degree of interaction of SAW or APW with a film placed on the waveguide surface is different from the case with a BAW resonator. Thus, it is necessary to develop appropriate technologies for creation of the films based on mycelium of higher fungi and their deposition on the surface of piezoelectric plates. As is known, there are several approaches to the creation of the aforementioned films [16,17]. For example, it is possible to use a homogenizate or mycelium extract with various concentrations, to include a polymer in the resulting film, to use ethanol, water, or water/ethanol mixtures with different ratios as solvents, etc. The most appropriate method for creation of the mycelium-sensitive film for acoustic delay line gas sensors is currently unknown. There is also no information about the degree of influence of one or another type of mycelium film on the properties of SAW or APW without gas affect. It is also unknown how the characteristics of SAW or APW will depend on the changes in the properties of the mycelium film as a result of interactions with a particular gas.

Thus, the purposes of the work were (i) to develop a technology and to create films based on mycelia of higher fungi which would introduce minimal losses in SAW or APW propagation at different frequencies, (ii) to elucidate the degree of influence of various vapors on the acoustic responces of the corresponding SAW and APW delay lines, and (iii) to analyze the temporal stability of the films obtained.

It should be noted that the choice of a higher fungus species for research is a difficult task. An important criterion in this case is the ability of the mycelium of the fungus to produce metabolites of various chemical compositions over the course of its life activity. Therefore, in this work, higher fungus *Ganoderma lucidum* (Curtis) P. Karst (Basidiomycota, Polyporaceae) was used. This fungus has high biosynthetic abilities like other basidiomycetes. *Ganoderma lucidum* (*G. lucidum)* metabolites are diverse in terms of chemical structure, physicochemical properties, and biological functions. The most well-known metabolites of *G. lucidum* have a beneficial effect on human and animal health. They exhibit antitumor, immunomodulatory, antiviral, antiallergic, antioxidant, neuroactive, or/and other properties [20,21,22,23]. *G. lucidum* is used to obtain various enzymes, including lignolytic enzymes, that are of the greatest interest [24]. There has been a known successful attempt to create composite materials based on *G. lucidum* [25].

## 2. Materials and Methods

### 2.1. Fabrication of the SAW and APW Devices for Gas Sensors

**Figure 1:**
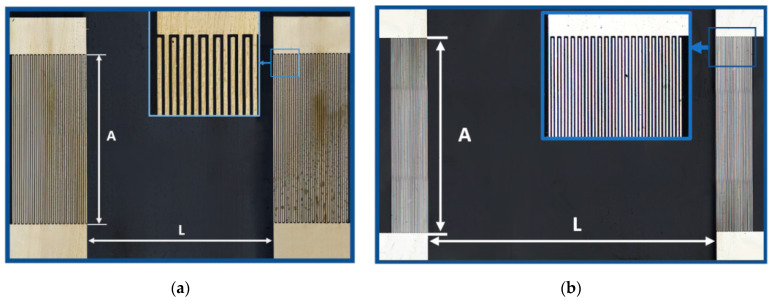
Optical images of the acoustic devices. (**a**) samples #1 and #2; (**b**) sample #3.

The characteristics of the acoustic devices exploited in the paper are presented in Table 1 and on Figure 1.

The substrate materials used for the devices are commercially available 128Y-*LiNbO*_3_ (Euler angles 0°, 37.86°, 0°) and YX-*LiNbO*_3_ (0°, 90°, 0°) wafers (Fomos Materials, Moscow, Russia) with a thicknesses of *h* = 305, 500, and 1900 μm. The plates have one grinded surface and one polished surface. The grinded surface (optical class Δ10) has averaged horizontal and vertical roughnesses of 0.16 μm and 0.8 μm, respectively. The polished surface (optical class Δ14) has averaged horizontal and vertical roughnesses of 0.01 μm and 0.05 μm, respectively. Two interdigital transducers (IDTs) were deposited on the polished surface in order to generate and to receive an acoustic wave. A sensitive film was located between the input and output IDTs in order to modify the propagation path and to improve vapor adsorption.

Each IDT comprises 20 finger electrodes patterned from 1000-nm-thick *Al* by means of projection maskless photolithography Smart Print (Microlight3D, La Tronche, France). The normalized substrate thickness for the acoustic plate waves (APWs) was *h*/*λ* = 305 μm/200 μm = 1.5 (sample #1). The normalized thickness of the substrates for the surface acoustic waves (SAWs) was about *h*/*λ* = 1900 μm/200 μm ≈ 500 μm/48 μm ≈ 10 (samples #2 and #3). This value provides a large number of modes along the propagation direction and their variety. A large number of electrodes pairs (20) ensures narrow pass band of the IDTs (5%) and good frequency resolution of the acoustic wave modes (±0.5 MHz) with close velocities *v_n_* (±200 m/s). 

Before IDTs puttering, the polished surface was cleaned with acetone and *Ar* plasma. Then, it was coated with *Al* film (400 nm) using a dc magnetron system VSE-PVD-DESK-PRO (LLC “Vacuum systems and electronics”, Novosibirsk, Russia) for ~4 min at discharge power of 200 W. The working pressure inside the chamber was 5.7 × 10^−3^ Torr. As the sample is bombarded by high energy particles and heated during the sputtering, it is cooled before leaving the chamber in *Ar* atmosphere for about 1 h.

As-fabricated *Al* film was coated with 2 μm thick photoresist (Heidolph MR Hei-Tec, Germany) using table-centrifuge, and tanned at 94 °C for 30 min (Heidolph MR Hei-Tec, Germany). Digital photo masks with two IDTs were fabricated using free-of-charge software (Layout). The mask was patterned on the sample coated with photoresist without any common photo mask (Smart Print, Microlight 3D, France). The exposure time was varied depending on the amplification of the objective strength. The exposed parts of photoresist were removed by developer P-236A-MF (FRAST-M, Moscow, Russia). The *Al* film located in the regions free of photoresist was etched with a mixture of orthophosphoric and nitric acids (95:5) for 60 min.

The optical images of the acoustic delay lines produced were obtained by means of a laser confocal microscope LEXT OLS 5000 (Olympus, Tokyo, Japan) (Figure 1). The samples #1 and #2 were fabricated using the same photo mask with parameters presented in Table 1. 

### 2.2. The Fungal Strain and Cultivation Conditions

The *G. lucidum* strain 5.1 from the collection of the Laboratory of Biosynthesis of Biologically Active Substances, Gauze Institute of New Antibiotics was used in this study. This strain was used in phylogenetic studies and the studied RNA sequence was deposited in the GenBank. Stock cultures were grown on potato glucose agar (PGA) at 26 °C for 7 days and then stored at 4 °C.

The liquid seed culture of *G. lucidum* was grown in 750 mL Erlenmeyer flasks containing 100 mL of liquid nutrient medium at 26 °C for 6 days. Unhopped beer wort (4° according to Balling scale) and pH 6.0 before sterilization was used as a liquid seed medium. The seed liquid medium was inoculated with mycelial agar plugs (3 mm diam.) of a seven-day culture of basidiomycete on PGA at the rate of 1/4 tube per flask. 

Submerged cultivation of *G. lucidum* was carried out on a rotary shaker at 220 rpm and at 26 °C in 750 mL Erlenmeyer flasks containing 100 mL of nutrient medium inoculated with 10 mL of liquid seed culture for 7 days. The liquid nutrient medium for submerged cultivation was contained (g/l of water): anhydrous glucose 20.0, yeast extract (Serva) 10.0, potassium dihydrogen phosphate 2.0, and magnesium sulfate 0.2. All ingredients of the culture medium used were water-soluble, which ensured that no non-fungal compounds presence in the target films. 

All nutrient media used in this work were sterilized at 1.2 atm for 30 min. The microbiological purity of cultures at all stages of work was controlled by using a light microscope BX41 (Olympus, Tokyo, Japan). The morphological characteristics of the submerged mycelium of the *G. lucidum* were observed by SEM using a high-resolution scanning electron microscope Mira II (Tescan, Warrendale, PA, USA).

**Figure 2:**
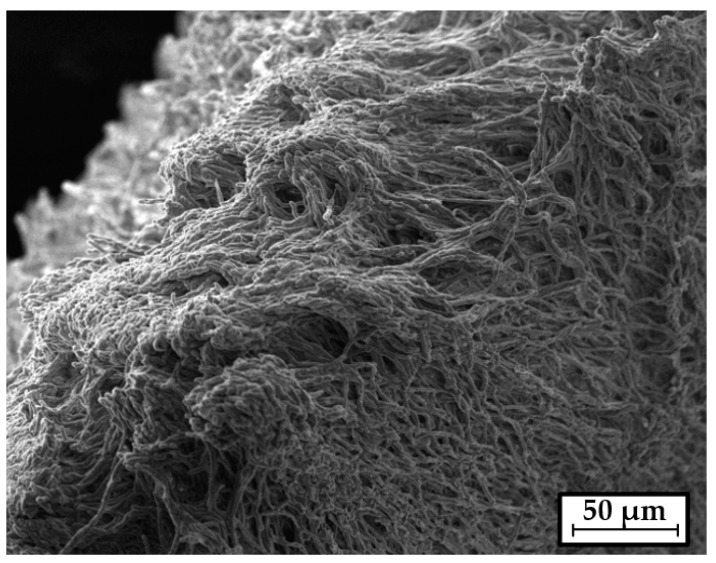
The SEM image of lyophilized submerged mycelium of *G. lucidum*.

After cultivation, the *G. lucidum* submerged mycelium was separated from the culture liquid by filtration, washed twice with distilled water, and lyophilized (Figure 2). The dry mycelium was ground with a T 25 ULTRA-TURRAX digital homogenizer (IKA, Staufen, Germany) at 10,000 rpm for 5 min in 48% aqueous ethanol. The ratio of mycelium to aqueous ethanol was 12.5 mg/mL. As a result, crushed mycelium of the *G. lucidum* in a solvent system (MEGl) was obtained. After that, the resulting suspension was centrifuged at 3500 rpm for 5 min and the supernatant was pipetted. As a result, the supernatant of a water/ethanol extract of the *G. lucidum* mycelium extract (EGl) was obtained. 

### 2.3. Technique for Producing Sensitive Films Based on Mycelium of Higher Fungi

**Figure 3:**
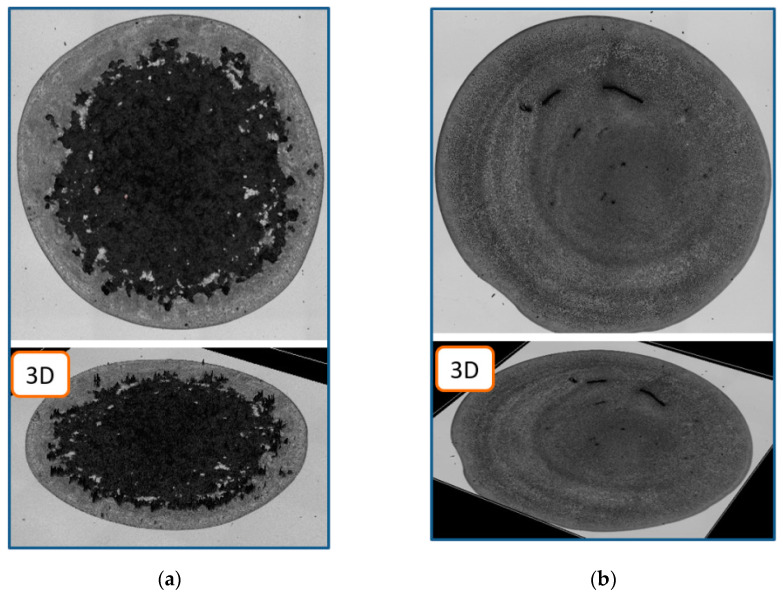
Images (**upper** row) and 3D visualizations (**lower** row) of the films based on (**a**) MEGl and (**b**) EGl obtained with a laser confocal microscope.

The films were obtained by using a Lenpipet Light dispenser (Thermo Fisher Scientific, Walthem, MA, USA) with a working volume of 1–10 μL. The volume of the initial drops during application and the drying time were 8 μL and 45 min, respectively. As a result, the average thickness of the films based on MEGl and EGl were 4.7 µm and 1.5 µm, respectively. The film area in both cases was 35 mm^2^. The images of the films based on MEGl and EGl obtained by means of a laser confocal microscope Lext OLS5000 (Olympus, Tokyo, Japan) at a laser scanning mode and their 3D visualization created by using the built-in software are shown in Figure 3. It can be seen that the MEGl film has a higher roughness than the EGl one. The higher roughness of the MEGl film, as well as its large thickness, is associated with the presence of fungal mycelium particles. It is possible to see that the MEGl film is more heterogeneous and rough than the EGl one. 

### 2.4. Deposition of Sensitive Films on Acoustic Delay Lines

**Figure 4:**
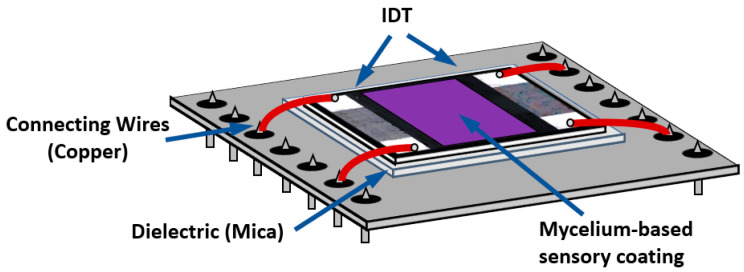
Schematic view of an acoustic delay line with sensitive mycelium film deposited.

The sensitive films between input and output IDTs were deposited using a removable rectangular mask. Then, the films were smoothed by means of a micro-brush. The dimensions of the masks were 7.5 × 14 mm^2^ (105 mm^2^) for samples #1 and #2, and 5 × 7 mm^2^ (35 mm^2^) for sample #3. The volume of the suspension was calculated depending on a square of the mask. For example, for 35 mm^2^, it was 8 µL for the MEGl and EGl films. Figure 4 shows the schematic view of an acoustic delay line with sensitive film deposited. 

### 2.5. Impact of the Mycelium Sensitive Films on the Performance of the Acoustic Devices

**Figure 5:**
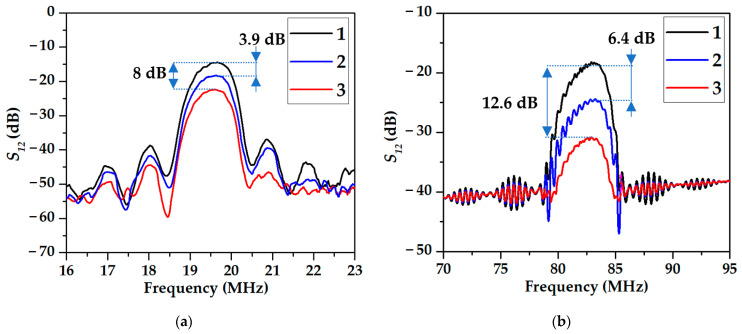
The frequency dependencies of the transfer functions S_12_ (insertion loss) of (**a**) SAW device #2 based on 128YX-LiNbO_3_ substrate with *f* = 19.7 MHz and (**b**) SAW device #3 based on YX-LiNbO_3_ substrate with *f* = 83 MHz with uncoated surface (1); with surface coated by EGl film (2), and with surface coated by MEGl film (3).

The frequency dependencies of the transfer functions *S*_12_ (insertion loss) of the SAW devices for 19.7 MHz (a) and 83 MHz (b) measured without any film (1), with EGl (2), and with MEGl (3) films deposited between IDTs are presented in Figure 5. It is seen that the presence of the MEGl film leads to a higher increase in *S*_12_ (8 dB at 19.7 MHz and 12.6 dB at 83 MHz) than for the EGl film (3.9 at 19.7 MHz and 6.4 dB at 83 MHz). This result is attributed to smaller thickness and lower roughness of the EGl film. Taking into account the different length of the sensitive films for devices #2 and #3 (10 and 7.5 mm, respectively), the maximum attenuation due to the film’s presence is less than 1 dB/mm, which is acceptable for acoustic measurements. The wave attenuation due to the presence of the films is evidently increased with the increase of the wave frequency.

**Figure 6:**
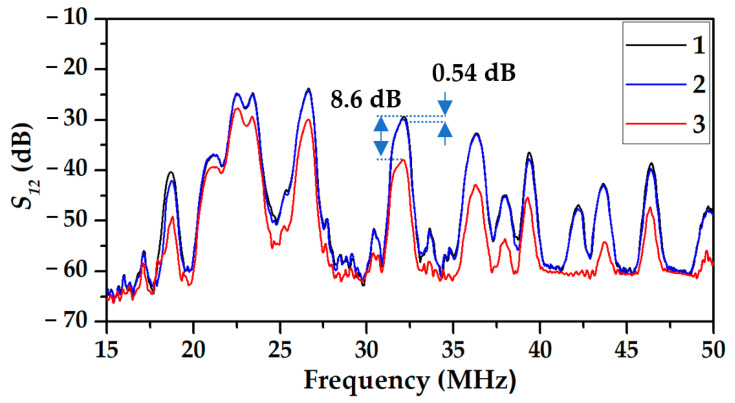
The frequency dependencies of the transfer functions *S*_12_ (insertion loss) of APW device #1 based on YX-*LiNbO*_3_ plate with with uncoated surface (1); with surface coated by EGl film (2), and with surface coated by MEGl film (3).

The same dependencies for device #1 based on APW are presented in Figure 6. Here, about 10 modes of zero and high orders are propagated simultaneously in the same direction. Each mode has its own frequency, amplitude, and response towards any action. In particular, the mode at the frequency of 32.2 MHz is one of the most sensitive towards loading the plate surface. The amplitude of this mode is decreased on 0.54 dB and 8.6 dB due to EGl and MEGl films deposition, respectively. It will be shown that this mode has high sensitivity towards some gases as well. 

### 2.6. Technique for Study of Gas-Sensitive Properties

**Figure 7:**
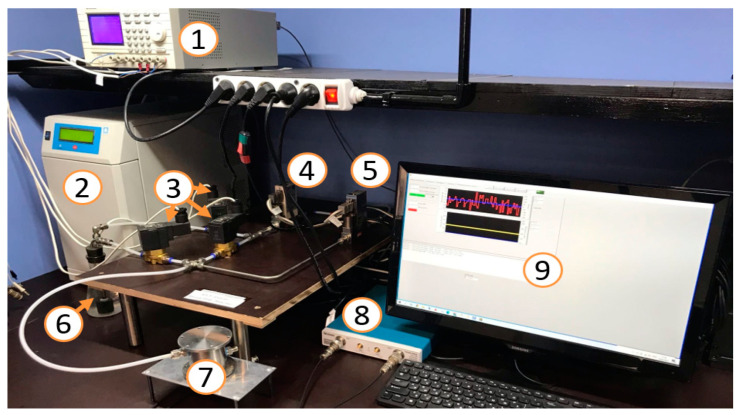
Photo of experimental setup for sensors testing. (1) 3-channel programmable power source; (2) dry air generator; (3) electromagnetic valves; (4) flow controller (Bronkhorst El-Flow Prestige); (5) flow controller (Bronkhorst El-Flow); (6) bubbler; (7) test chamber; (8) network analyzer (Tektronix TTR 506A); (9) PC with MOXA interface card.

**Figure 8:**
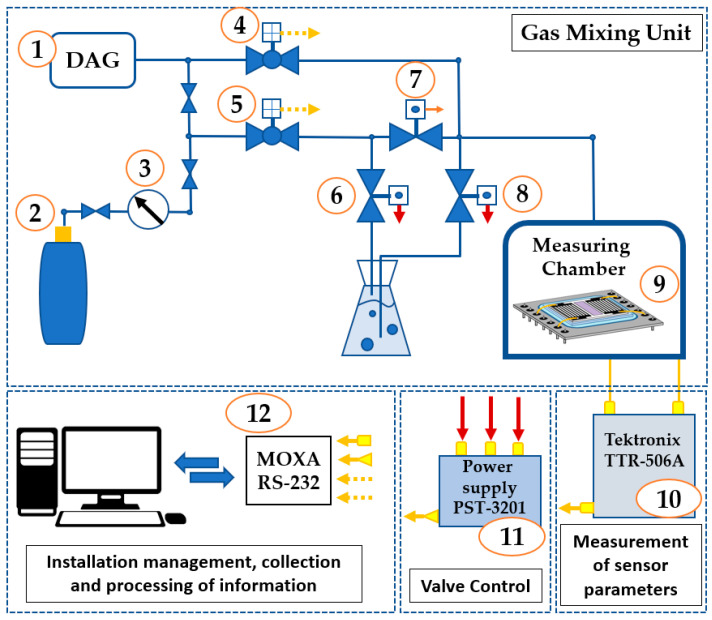
Scheme of the experimental setup for sensors testing. (1) dry air generator-DAG; (2) test gas cylinder; (3) pressure regulator; (4) flow controller (Bronkhorst El-Flow); (5) flux controller Bronkhorst (El-Flow Prestige); (6), (7), (8) electromagnetic valves; (9) measuring chamber; (10) network analyzer (Tektronix TTR 506A); (11) 3-channel programmable power source; (12) PC with MOXA interface card.

The photo and scheme of the experimental set up used for study of the sensing properties of the gas sensors produced are presented in Figure 7 and Figure 8, respectively.

The operation of the setup used is controlled by software based on LabView. Software allows set gas flows in accordance with programmed recipe, to open and to close the valves, to set frequency of an acoustic wave, and to write measured values to file. The gas-mixing system consists of units depicted on the scheme as (1)–(8). The air source (1) is supplying dried and catalytically purified air. Gas cylinder (2), through pressure regulator (3), is connected to flow controller (4) (Bronkhorst El-Flow Prestige, 5–500 mL/min) calibrated for dry air. Electromagnetic valves (6)–(8) are controlled by 3-channel programmable power source (11), switching a test gas flow from gas cylinder to bubbler. As a result, a test air–vapor mixture (vapors of ethanol, acetone, and water) was prepared by means of bubbling of dry air through a liquid substance. The test chamber (9) is made of stainless still and its input and output are connected to gas-mixing system and ambient air, respectively. A network analyzer TTR 506A (Tektronix, Beaverton, OR, USA) (10) was used for measurement of characteristics of the sensor under study. The setup monitoring, measured data collecting, and processing were provided by RS-232 MOXA interface card.

### 2.7. Calculation of the Test Vapor Concentration in Gas Flow

**Figure 9:**
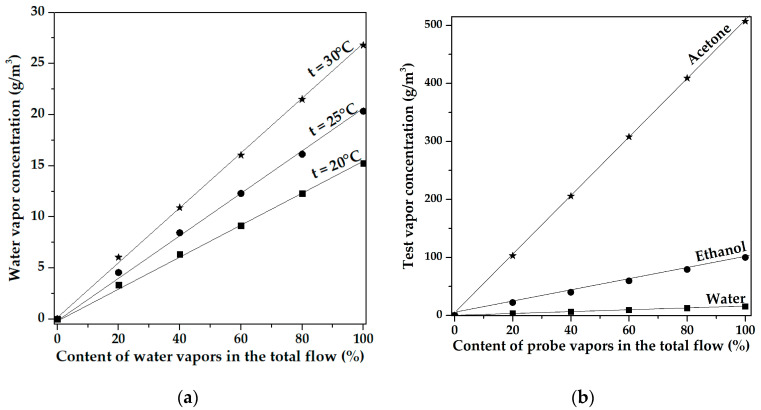
(**a**) Water vapor concentration vs. content of water vapors in the total gas flow at temperature 20 °C, 25 °C, and 30 °C; (**b**) test vapor concentration vs. content of probe vapors in the total gas flow at temperature 20 °C.

The rate of a total gas flow through the test chamber was constant (100 mL/min). The test vapor concentration in the total gas flow was changed by varying the ratio between the dry air and test vapor flows through the bubbler. The temperature and the humidity inside the chamber were monitored by thermo-hygrometer IPVT-03-01-2V (Eksis, Zelenograd, Russia), with accuracies of ±0.2 °C and ±2%, respectively. The next procedure was used to determine the concentration of ethanol and acetone vapors in the total gas flow due to the lack of the calibrated sensors for these vapors. First, the concentration values (*C_g_*) of distilled water vapor using an IPVT-03-01-2V thermohygrometer were determined for different ratios of dry and humid air flows at different temperatures (20 °C, 25 °C, 30 °C). These dependencies are shown in Figure 9a and corresponding values are presented in Table 2 [26]. 

As is known, totally saturated vapors could not be obtained by bubbling. In this case the level of saturation was estimated by using the Mendeleev–Claperon Equation (1):

(1) $$C_{g}=\frac{P_{s}}{k*T}*\frac{M}{N_{A}}$$

where *C_g_* is the gas concentration, *P_S_* is saturated vapor pressure, *k* is the Boltzmann constant, *T* is the absolute temperature, *M* is molar mass, and *N_A_* is the Avogadro’s number. As can be seen from Figure 9a, with an undiluted flow of humid air, the concentration of water vapor is equal to 15.39 g/m^3^, 20.63 g/m^3^, and 26.96 g/m^3^ at 20 °C, 25 °C, and 30 °C, respectively. As was calculated by means of (1), the concentration of saturated water vapor is equal to 17.23 g/m^3^, 23.17 g/m^3^, and 30.5 g/m^3^ at temperatures of 20 °C, 25 °C, and 30 °C, respectively. Thus, the maximum concentration of water vapor (100%) in the test sample flow entering in the chamber corresponds to 89.4%, 89.6%, and 89.1% of the concentration of saturated water vapor calculated by using (1) at 20 °C, 25 °C, and 30 °C, respectively. Assuming the validity of the procedure described above for ethanol and acetone, it was supposed that the concentration of these vapors after bubbling was also about 89.1–89.6% of the relevant saturated values. The corresponding concentrations of the vapors for various flow ratio test vapor/dry air at t = 20 °C are presented in Figure 9b and Table 2. 

The concentrations for saturated vapors of acetone and ethanol calculated by using (1) are equal to 586.98 and 111.49 g/m^3^, respectively. Taking into account the above estimates for water vapor, these values were multiplied by 0.89. As a result, it was obtained that the concentrations of saturated vapors of ethanol and acetone are equal to 99.81 g/m^3^ and 507.25 g/m^3^, respectively (Table 2). Other concentrations for ethanol and acetone vapors were obtained from linear approximation (Figure 9b).

### 2.8. Repeatability of the Results Measured

**Figure 10:**
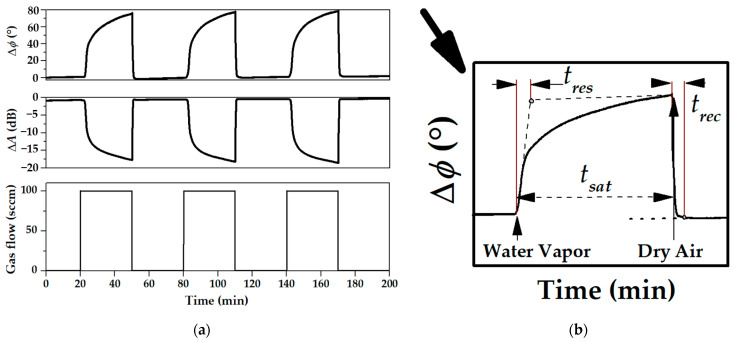
Phase (**a**) Time dependencies of phase and amplitude responses of the APW device #1 (*f* = 32.2 MHz) towards water vapor measured for three cycles of the vapor injections for RH = 89.4% at *t* = 20 °C; (**b**) schematic presentation of the phase response for definition of *t_res_*, *t_sat_*, and *t_rec_* sourced from (**a**) (upper row).

Repeatability of the measurements was studied for all acoustoelectronic devices and test vapors examined in the paper. As an example, Figure 10a shows the time dependencies of the phase (∆*ϕ*) and amplitude (∆*A*) responses of the APW device #1 with the MEGl film towards water vapor measured at 32.2 MHz and RH = 89.8%. It is seen that the values of time response are very close each other for all cycles of vapor injections. It will be useful to distinguish time of response (*t_res_*) and time of saturation (*t_sat_*). The definition of these parameters on the example of a single time-phase response is presented in Figure 10b. Here, *t_res_* is determined as a point of intersection of the tangents to the curve sections at the initial and final stages of the time response. It should be noted that values of *t_res_* and recovery time (*t_rec_*) are very close and equal to 140–150 s, but the *t_sat_* is about 30 min.

### 2.9. Method for Calculation of the Acoustoelectronic Devices Responses

In order to properly compare the responses from acoustoelectronic devices differing in IDT period, material and geometric dimensions of the film, wave type, and distance between IDTs, the following approach was used.

**Figure 11:**
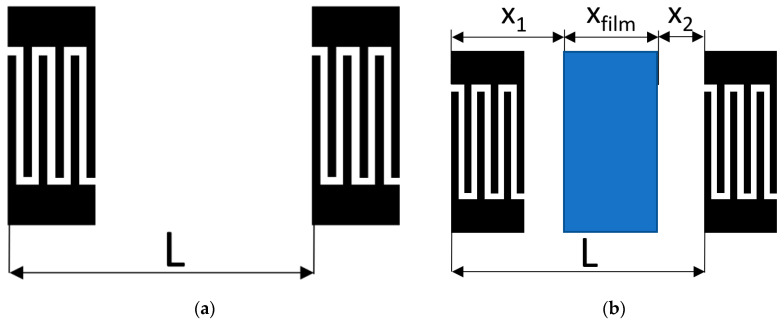
Geometries of acoustic SAW and APW delay lines used for comparing the measured gas responses. (**a**) without film; (**b**) with film.

First, the change in insertion loss ∆*S*_12_*^uncoated^* produced by a gas adsorption is measured for uncoated sample in geometry of Figure 11a, when the propagation path having length L does not contain any film. Then, the same response ∆*S*_12_*^coated^* is measured for the coated sample in geometry of Figure 11b when part of the propagation path (*X*_1_ + *X*_2_) is uncoated and the other part (*X_film_*) contains a sensitive film: ∆*S*_12_*^film^* = ∆*S*_12_*^coated^* − ∆*S*_12_*^uncoated^*(*X*_1_+ *X*_2_)/*L*. Here ∆*S*_12_*^coated^* and ∆*S*_12_*^uncoated^* are measured, while *X*_1_, *X*_2_, *L* are known. As a result, the amplitude response for a film towards a vapor injection for SAW and APW is:

α = ∆*S*_12_*^film^/X_film_* [dB/mm]. (2)

Similarly, the phase response for a film is:

(3) $$R=∆\varphi^{film}/360^{0}\left(X_{film}/\lambda \right),\;\left[\mathrm{ppm}\right]$$

where ∆*φ^film^* = ∆*φ^coated^* − ∆*φ ^uncoated^* (*X*_1_+ *X*_2_)/*L*, ∆*φ^coated^* and ∆*φ ^uncoated^* are measured, *X*_1_, *X*_2_, *X_film_*, *L* are known, and 360° (*X_film_*/*λ*) is the total phase acquired a wave, propagating along a film of a length *X_film_*.

## 3. Results and Discussion

The maximum yield of the *G. lucidum* mycelium on a liquid nutrient medium with glucose and peptone reached 10.0 ± 0.3 g/l on the 7th day of submerged cultivation. The hyphal structure of the mycelium used is retained in the lyophilized state (Figure 2).

**Figure 12:**
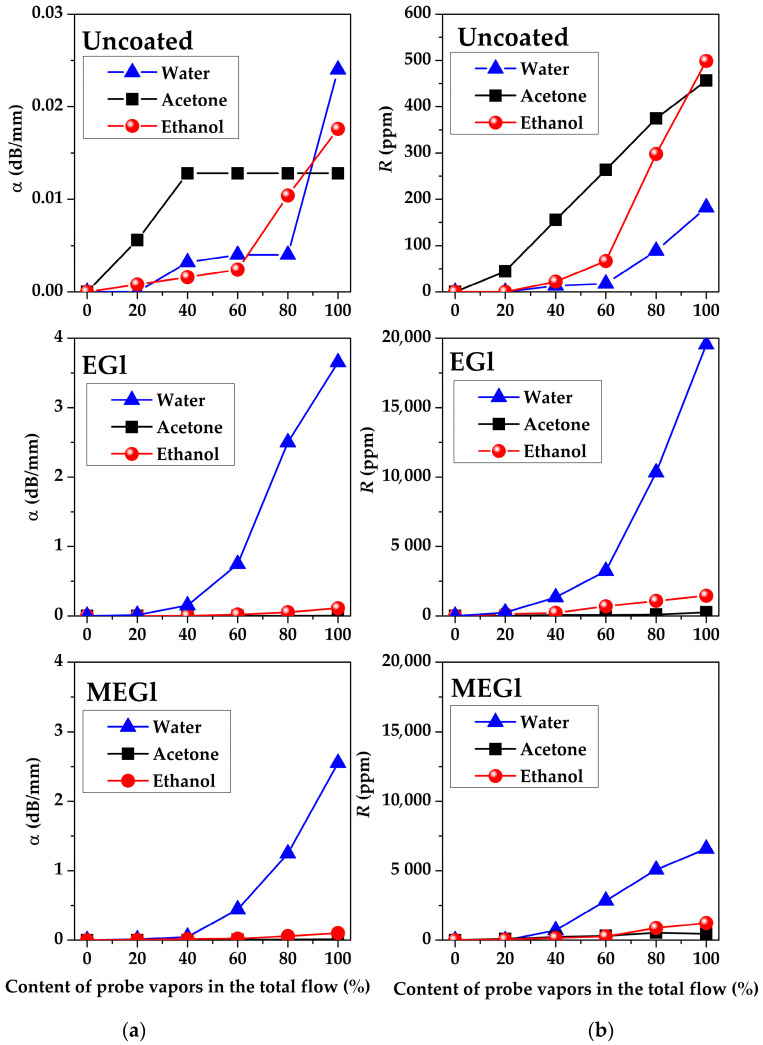
The dependencies of (**a**) amplitude *α* and (**b**) phase *R* responses of SAW delay line #2 (128°YX-*LiNbO_3_*, *f* = 19.7 MHz) towards water, acetone, and ethanol vapors measured for uncoated (**upper** row), EGl coated (**middle** row), and MEGl coated (**lower** row) substrates on vapor concentrations.

**Figure 13:**
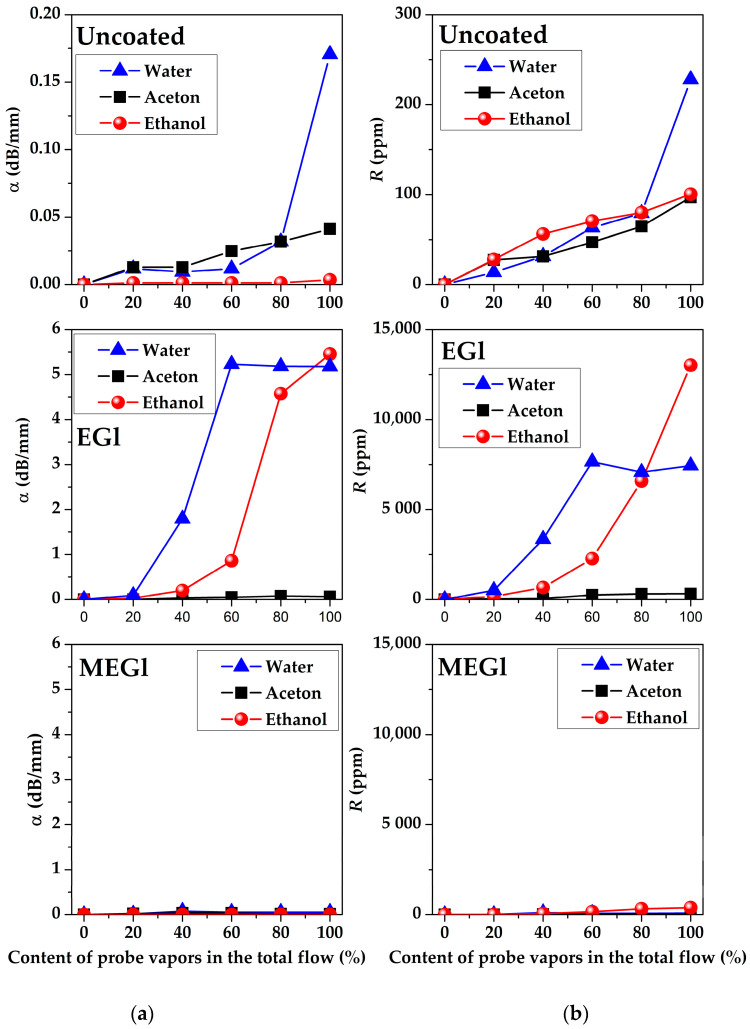
The dependencies of (**a**) amplitude *α* and (**b**) phase *R* responses of SAW delay line #3 (YX-*LiNbO_3_*, *f* = 83 MHz) towards water, acetone, and ethanol vapors measured for uncoated (**upper** row), EGl coated (**middle** row), and MEGl coated (**lower** row) substrates on vapor concentrations.

**Figure 14:**
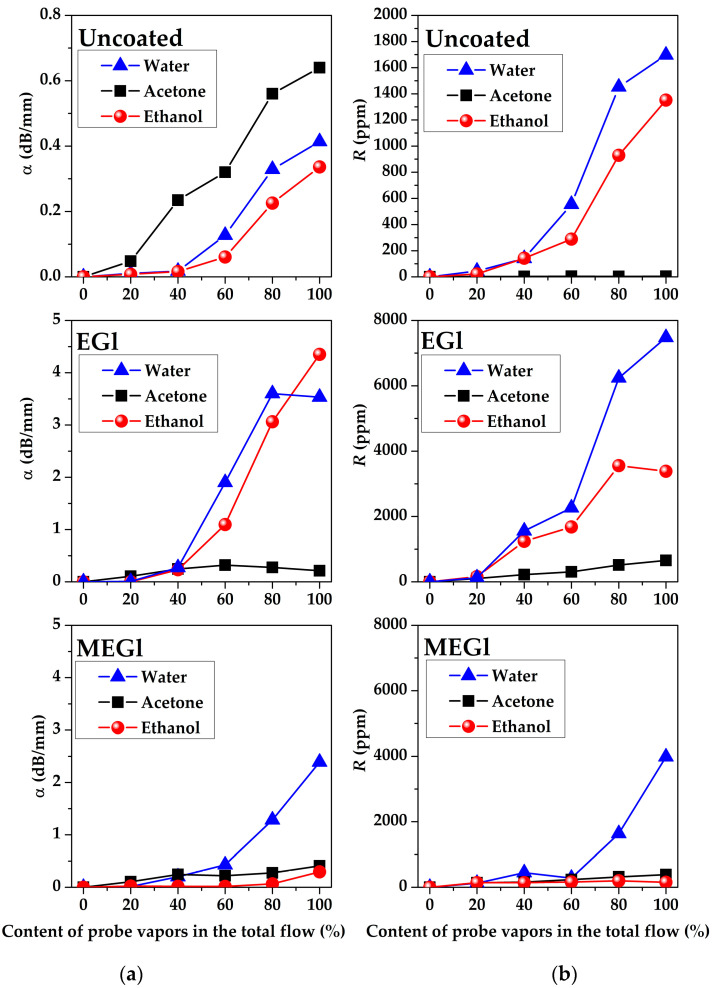
The dependencies of (**a**) amplitude *α* and (**b**) phase *R* responses of APW delay line #1 (YX-*LiNbO_3_*, *f* = 32.2 MHz) towards water, acetone, and ethanol vapors measured for uncoated (**upper** row), EGl-coated (**middle** row), and MEGl-coated (**lower** row) substrates on vapor concentrations.

The dependencies of the amplitude α and phase R responses of all acoustoelectronics delay lines #1, #2, and #3 towards water, acetone, and ethanol vapors measured for uncoated, EGl-, and MEGl-coated substrates on vapor concentrations are presented in Figure 12, Figure 13 and Figure 14. It is possible to see that these dependencies demonstrate big differences from one another depending on substrate material, film and vapor types, wave type and frequency, and vapor concentration. The structure of the films is more or less inhomogeneous and contains pores and gaps. We assume that the process of gas absorption by such films is of a physical rather than chemical nature. That is why the restoration of film characteristics after removal of the gas is observed. The process of physical absorption is accompanied by two effects. First, gas molecules are adsorbed on the surface of the film and its mass increases. This leads to a decrease in the velocity of acoustic waves and an increase in their attenuation. Second, gas molecules penetrate from the surface of the film into its volume. This leads to a change in its moduli of elasticity. In this case, the velocity and attenuation of acoustic waves can either increase or decrease depending on the ratio of BAW velocities in the film and substrate material. Both the mass and elastic effects depend on the film material, its microstructure, and the type of vapor. In experiments, acoustic responses to any vapor depend on a combination of these effects. 

Figure 12 shows that the amplitude *α* and phase *R* responses measured for SAW delay line #2 at f = 19.7 MHz with any film used are higher than ones for uncoated substrate for all test vapors. The response to all vapors used for a SAW delay line #2 without sensitive film does not exceed 0.03 dB/mm in amplitude and 500 ppm in phase. Responses to water vapor are dominant for both types of films used. It is possible to see that the EGl film-coating counterpart is more sensitive than that of the MEGl film. The largest responses *α* = 3.8 dB/mm and *R* = 19500 ppm were obtained for water vapors in the presence of the EGl film at RH ≈ 89.4%. It can be seen that the magnitude of the response to water vapor at their maximum concentration increases more than 150 times in amplitude (from 0.25 to 3.8 dB/mm) and more than 110 times in phase (from 170 to 19500 ppm). The responses to acetone practically do not change and amount to *α* = 0.15 dB/mm and *R* = 450 ppm both for the device without sensitive film and for the device with EGl and MEGl films. The response to ethanol vapor in phase increases approximately twofold from 500 to 1000 ppm, and approximately 5–6 times in amplitude from 0.15 to 0.1 dB/mm.

Figure 13 shows that the amplitude α and phase R responses measured for SAW delay line #3 at *f* = 83 MHz coated with EGl film is higher than ones for uncoated and MEGl film coated substrates. As shown above (Figure 5b), in the absence of any vapors, the MEGl film introduces more significant losses in the acoustic signal (12.5 dB) than the EGl film (6.4 dB). This is due to the fact that the MEGl film is more rough than the EGl film.

In this regard, the value of the response of the acoustic signal to the test vapors effect in the case of the MEGl film is much lower than for the EGL film at higher frequencies. The EGl film introduces less attenuation into the vapor-free acoustic signal at higher frequencies (delay line #3), and the response to water vapor in this case is comparable to the response of delay line #2 (*f* = 19.7 MHz) with the MEGl coating (Figure 12). At the same time, for the SAW delay line #3 (*f* = 83 MHz), a higher response is observed to ethanol vapor than to water or acetone vapor. It is possible to see that for device #3 with the EGl film, the largest responses are equal to *α* = 5.2 dB/mm and *R* = 13000 ppm for maximal concentration of ethanol vapor in total gas flow (Figure 13). Thus, it is possible to make the conclusion that MEGl film is not appropriate for high frequency SAW sensor as a sensitive coating.

On the contrary, the responses of APW delay line (*f* = 32.19 MHz) for uncoated and coated substrates towards water and ethanol vapors are more or less comparable with each other (Figure 14). 

In general, the EGl film looks more sensitive than the MEGl film to all vapors. This is most likely due to the fact that the MEGl film on the surface of the APW delay line results in more acoustic signal propagation loss than the EGl film (Figure 6). The largest responses *α* = 4 dB/mm and *R* = 7500 ppm are obtained for device #1 with EGl film to the maximum concentration of ethanol vapors in the total gas flow.

The analysis of the results obtained has shown that both films, regardless of the type of wave and frequency, weakly react to acetone vapor. Perhaps this is due to the lack of affinity between the acetone molecules and the low molecular weight compounds present in the mycelium of the fungus *G. licudum.*

Comparison of the SAW and APW devices performed only using Figure 12, Figure 13 and Figure 14 is very preliminary because the waves have different frequencies and because the APW sensing is varied with mode order, plate thickness, film thickness, and wavelength, while it is not optimized in the paper. Nevertheless, it may surely be concluded, even though the waves belong to different types and have different concentration near the sensing surface, they may have comparable gas sensitivity, in general.

**Figure 15:**
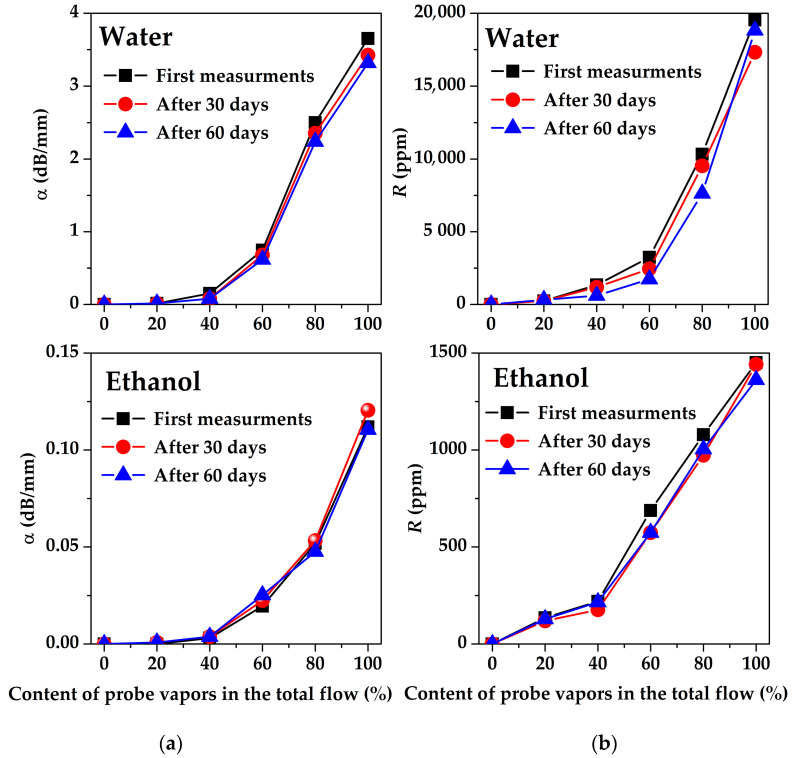
The dependencies of (**a**) the amplitude *α* and (**b**) the phase *R* responses of the SAW delay line #2 (128°YX-*LiNbO*_3_, *f* = 19.7 MHz) with EGl film towards water (**upper** row), and ethanol (**lower** row) vapors on vapor concentrations measured during 60 days.

The long-term stability of all sensitive films was examined for 60 days with step 30 days. As an example, the amplitude *α* and phase *R* responses of SAW device #2 measured for fresh and aged EGl films are presented in Figure 15. It is seen that the results of the measurements are practically unchanged with time for all test vapors and their concentrations. Good long-term stability was shown to be inherent for other sensitive films as well.

## 4. Conclusions

Biological substances like films based on mycelium of higher fungi are suitable as sensitive films for acoustic wave gas sensors. In this work, higher fungus *Ganoderma lucidum* (Curtis) P. Karst (Basidiomycota, Polyporaceae) was used as a source of mycelium films. This fungus has high biosynthetic abilities like other basidiomycetes. *G. lucidum* metabolites are diverse in terms of chemical structure, physicochemical properties, and biological functions. The performed studies revealed that most suitable-for-film mycelium production serve the supernatant of a water/ethanol extract of *G. lucidum* mycelium extract (EGl). These films are compatible with acoustic wave technologies, produce small acoustic attenuation (up to 1 dB/mm), enhance the values of the gas responses as compared with uncoated substrates, and demonstrate good reproducibility and long-term stability. It is necessary to say that structure of the films used is inhomogeneous and contains pores and gaps. We assume that the process of gas absorption by such films is of a physical rather than chemical nature. That is why the restoration of film characteristics after removal of the gas is observed. The process of physical absorption is accompanied by two effects. First, gas molecules are adsorbed on the surface of the film and its mass increases. This leads to a decrease in the velocity of acoustic waves and an increase in their attenuation. Second, gas molecules penetrate from the surface of the film into its volume. This leads to a change in its moduli of elasticity. In this case, the velocity and attenuation of acoustic waves can either increase or decrease depending on the ratio of BAW velocities in the film and substrate material. Both the mass and elastic effects depend on the film material, its microstructure, and the type of gas or vapor. In experiments, acoustic responses to any vapor depend on a combination of these effects.

Based on the results obtained, it was concluded that MEGl film is not appropriate for use in high frequency SAW delay lines as a sensitive coating. However, both types of films (MEGl and EGl) could be used as sensitive coatings for low frequency SAW and APW sensors based on corresponding delay lines.

In this study, the selective films to test vapors (water, ethanol, and acetone) were not obtained. It was found only that both films, regardless of the type of wave and frequency, weakly react to acetone vapor. Perhaps this is due to the lack of affinity between acetone molecules and low molecular weight compounds present in the mycelium of the fungus *G. licudum*. This task demands additional investigations. It is necessary to note that the MEGl and EGl films used in this paper were obtained from a water/ethanol solution. Fungal metabolites extracted by this solvent system can vary significantly in their hydrophilicity/hydrophobicity. The affinity of hydrophilic metabolites of *G.lucidum* for compounds in the series water, ethanol, acetone will decrease, and the affinity of hydrophobic metabolites for these compounds will increase. Therefore, it is highly likely that different compounds interact with each of the three studied vapors. The use of a water/ethanol extract of *G. lucidum* mycelium does not allow one to achieve film selectivity due to the simultaneous presence of metabolites interacting with different vapors. To achieve selectivity, it is necessary to isolate fractions or individual metabolites of *G.lucidum* that show affinity for only one of the gases. Thus, the problem of obtaining a selective sensitive coating based on such mycelium films for SAW and APW gas sensors will be solved in further stages of work.

In whole, the performed work has shown that the improvement of the sensitive properties and selectivity of acoustic gas sensors based on fungal mycelium is possible by exploiting the substrate anisotropy, variation of operation frequency, plate thickness, order of the wave modes, chemical composition, and structural properties of mycelium film.

## Figures and Tables

**Table 1:** Parameters of acoustic devices used.

Sample #	Wave Type	Plate Orientation	Plate Thickness *h*, μm	Distance between IDT *L*, mm	Aperture *A*, mm	Number of IDT Pairs	Wavelength *λ*, μm	Freq. *f*, MHz
1	Plate	YX-*LiNbO*_3_	305	10	8.5	20	200	32.2
2	SAW	128°YX-*LiNbO*_3_	1900	10	8.5	20	200	19.7
3	SAW	YX-*LiNbO*_3_	500	7.5	5	20	48	83

**Table 2:** Vapor concentrations via flows ratio (t = 20 °C).

Flow Ratio Test Vapor/Dry Air	Water *C_g_*, g/m^3^ Measured	Ethanol *C_g_*, g/m^3^ Estimated	Acetone *C_g_*, g/m^3^ Estimated
20/80	3.26	21.96	102.75
40/60	6.25	39.92	205.63
60/40	9.12	59.56	307.95
80/20	12.27	78.98	408.94
100/0	15.39	99.81	507.25

## Data Availability

No new data were created or analyzed in this study. Data sharing is not applicable to this article.

## References

[1] Kumar A., Prajesh R. (2022). The potential of acoustic wave devices for gas sensing applications. Sens. B Actuators A Phys..

[2] Anisimkin V. (2015). Optimization of an integrated lattice of acoustoelectronic gas sensor. J. Commin. Technol. Electron..

[3] Ballantine D.S., White R.M., Martin S.I., Ricco A.I., Zellers E.T., Frye G.C., Wohltjen H. (1997). Acoustic Wave Sensors: Theory, Design, and Physico-Chemical Application.

[4] Nazemi H., Joseph A., Park J., Emadi A. (2019). Advanced micro-and nano-gas sensor technology: A review. Sensors.

[5] Kuchmenko T.A., Lvova L.V. (2019). A perspective on recent advances in piezoelectric chemical sensors for environmental monitoring and foodstuffs analysis. Chemosensors.

[6] Constantinoiu I., Viespe C. (2021). Synthesis methods of obtaining materials for hydrogen sensors. Sensors.

[7] Afzal A., Iqbal N., Mujahid A., Schirhagl R. (2013). Advanced vapor recognition materials for selective and fast responsive surface acoustic wave sensors: A review. Anal. Chim. Acta.

[8] Gautam Y.K., Sharma K., Tyagi S., Kumar A., Singh B.P. (2021). Applications of green nanomaterials in coatings. Green Nanomaterials for Industrial Applications.

[9] Vidhya S., Murari B.M. (2016). Sol-gel thin film based sensors and biosensors. Int. J. Pharma Bio Sci..

[10] Zagnoni M. (2012). Miniaturised technologies for the development of artificial lipid bilayer systems. Lab Chip.

[11] Tehrani Z., Whelan S.P., Mostert A.B., Paulin J.V., Ali M.N., Ahmadi E.D., Graeff C.F.O., Guy O.J., Gethin D.T. (2020). Printable and flexible graphene pH sensors utilising thin film melanin for physiological applications. 2D Mater..

[12] Cejpkova J., Gryndler M., Hrselova H., Kotrba P., Randa Z., Synkova I., Borovicka J. (2016). Bioaccumulation of heavy metals, metalloids, and chlorine in ectomycorrhizae from smelter-polluted area. Environ. Pollut..

[13] Huang H., Cao L., Wan Y., Zhang R., Wang W. (2012). Biosorption behavior and mechanism of heavy metals by the fruiting body of jelly fungus (Auricularia polytricha) from aqueous solutions. Appl. Microbiol. Biotechnol..

[14] Kuznetsova I.E., Zaitsev B.D., Shikhabudinov A.M., Tsivileva O.M., Pankratov A.N., Verona E. (2017). Acousto-electronic Gas Sensor Based on Mushroom Mycelial Extracts. Sens. Actuators B.

[15] Silina Y.E., Kuchmenko T.A., Korenman Y.I., Tsivileva O.M., Nikitina V.E. (2005). Use of a complete factorial experiment for designing a gas sensor based on extracts of *Pleurotus ostreatus* mycelium mushroom. J. Anal. Chem..

[16] Kuznetsova I.E., Zaitsev B.D., Krasnopolskaya L.M., Teplykh A.A., Semyonov A.P., Avtonomova A., Ziangirova M.Y., Smirnov A.V., Kolesov V.V. (2020). Influence of humidity on the acoustic properties of mushroom mycelium films used as sensitive layers for acoustic humidity sensors. Sensors.

[17] Smirnov A.V., Asafiev N.O., Sorokin B.P., Ziangirova M.Y., Golyshkin A.V., Krasnopolskaya L.M., Kuznetsova I.E. (2020). Investigation of the influence of sensor films made of the mycelium of basidiomycetes on the characteristics of a Me1/AlN/Me2/diamond ulytahigh-frequency resonator. J. Commun. Technol. Electron..

[18] Grabka M., Witkiewicz Z., Jasek K., Piwowarski K. (2022). Acoustic wave sensors for detection of blister chemical warfare agents and their simulants. Sensors.

[19] Kuznetsova I.E., Anisimkin V.I., Gubin S.P., Tkachev S.V., Kolesov V.V., Kashin V.V., Zaitsev B.D., Shikhabudinov A.M., Verona E., Sun S. (2017). Super High Sensitive Plate Acoustic Wave Humidity Sensor based on Graphene Oxide Film. Ultrasonics.

[20] Almyasheva N.R., Yarina M.S., Golyshkin A.V., Dzhavakhyan B.R., Krasnopolskaya L.M. (2017). Antioxidant properties of water-soluble polysaccharides and ethanolic extracts of xylotrophic basidiomycetes mycelium. Antibiot. I Khimioterapiya.

[21] Cör D., Knez Ž., Hrnčič M. (2018). Antitumour, antimicrobial, antioxidant and antiacetylcholinesterase effect of *Ganoderma lucidum* terpenoids and polysaccharides: A Review. Molecules.

[22] Lianga C., Tiana D., Liua Y., Lia H., Zhub J., Lic M., Xind M., Xia J. (2019). Review of the molecular mechanisms of *Ganoderma lucidum* triterpenoids: Ganoderic acids A, C2, D, F, DM, X and Y. Eur. J. Med. Chem..

[23] Shankar A., Sharma K.K. (2022). Fungal secondary metabolites in food and pharmaceuticals in the era of multi-omics. Appl. Microbiol. Biotechnol..

[24] Sharma A., Jain K.K., Srivastava A., Shrivastava B., Thakur V.V., Jain R.K., Kuhad R.C. (2019). Potential of in situ SSF laccase produced from *Ganoderma lucidum* RCK 2011 in biobleaching of paper pulp. Bioprocess Biosyst. Eng..

[25] Sydor M., Cofta G., Doczekalska B., Bonenberg A. (2022). Fungi in Mycelium-Based Composites: Usage and Recommendations. Materials.

[26] Gorbachev I., Smirnov A., Ivanov G., Avramov I., Datsuk E., Venelinov T., Bogdanova E., Anisimkin V., Kolesov V., Kuznetsova I. (2023). Langmuir-Blodgett films of arachidic and stearic acids as sensitive coatings for chloroform HF SAW sensors. Sensors.

